# Saliva cortisol diurnal variation and stress responses in term and preterm infants

**DOI:** 10.1136/archdischild-2021-321593

**Published:** 2022-03-07

**Authors:** David Q Stoye, James P Boardman, Clive Osmond, Gemma Sullivan, Gillian Lamb, Gill S Black, Natalie Z M Homer, Nina Nelson, Elvar Theodorsson, Evalotte Mörelius, Rebecca M Reynolds

**Affiliations:** 1 MRC Centre for Reproductive Health, The University of Edinburgh, Edinburgh, UK; 2 Centre for Clinical Brain Sciences, The University of Edinburgh, Edinburgh, UK; 3 MRC Lifecourse Epidemiology Unit, University of Southampton, Southampton, UK; 4 Centre for Cardiovascular Science, The University of Edinburgh, Edinburgh, UK; 5 Department of Clinical and Experimental Medicine, Linköping University, Linkoping, Sweden; 6 Department of Neurobiology, Care Sciences and Society, Karolinska Institute, Stockholm, Sweden; 7 Division of Clinical Chemistry, Department of Biomedical and Clinical Sciences, Faculty of Medicine and Health Sciences, Linköping University, Linkoping, Sweden; 8 School of Nursing and Midwifery, Edith Cowan University, Joondalup, Western Australia, Australia

**Keywords:** neonatology, endocrinology, infant development

## Abstract

**Objective:**

To determine if preterm birth is associated with adaptation of the hypothalamic–pituitary–adrenal (HPA) axis and whether HPA axis programming relates to the degree of prematurity (defined as extremely preterm birth at <28 weeks or very preterm birth at 28–32 weeks gestation).

**Design:**

This study reports findings from a prospective birth cohort. Saliva cortisol concentrations were measured prevaccination and postvaccination, and in the morning and evening, at 4 months chronological age.

**Setting:**

Infants born at a single Scottish hospital.

**Participants:**

45 term-born, 42 very preterm and 16 extremely preterm infants.

**Outcomes:**

Cortisol stress response to vaccination (postvaccination minus prevaccination cortisol concentrations), diurnal slope (log-transformed morning minus log-transformed evening cortisol values) and mean log-transformed daily cortisol.

**Results:**

Compared with infants born at term, infants born extremely preterm had a blunted cortisol response to vaccination (5.8 nmol/L vs 13.1 nmol/L, difference in means: −7.3 nmol/L, 95% CI −14.0 to −0.6) and a flattened diurnal slope (difference in geometric means: −72.9%, 95% CI −87.1 to −42.8). In contrast, the cortisol response to vaccination (difference in means −2.7 nmol/L, 95% CI −7.4 to 2.0) and diurnal slope at 4 months (difference in geometric means: −33.6%, 95% CI −62.0 to 16.0) did not differ significantly in infants born very preterm compared with infants born at term.

**Conclusions:**

Infants born extremely preterm have blunted cortisol reactivity and a flattened diurnal slope. These patterns of HPA axis regulation are commonly seen after childhood adversity and could contribute to later metabolic and neurodevelopmental phenotypes observed in this population.

## Abstract (precis)

Results from salivary cortisol concentrations of infants at 4 months of age suggests that extremely preterm birth disrupts the normative development of the HPA axis in infancy, with patterns of cortisol secretion resembling those seen after childhood adversity.

**What is already known on this topic?**

- Preterm birth is associated with adverse metabolic and neurodevelopmental phenotypes across the life course.
- Hypothalamic–pituitary–adrenal axis (HPA) dysregulation has been observed following multiple types of prenatal and childhood adversity and is a potential biological mediator of later pathology.

**What this study adds?**

- At 4 months postnatal age extremely preterm birth is associated with a blunted cortisol response to vaccination and a flattened diurnal rhythm.
- Future studies investigating whether HPA axis adaptations after preterm birth continue across the life course and relate to adverse neurodevelopmental and metabolic phenotypes appear warranted.

## Introduction

Preterm birth (<37 weeks gestation) is associated with an increased risk of adverse health across the life course, including neurodevelopmental impairment,^1 2^ inattention, mood disorders and psychosis,^3^ metabolic disorders,^4^ and cardiovascular disease.^5^ However, the biological mechanisms linking preterm birth with future morbidity remain largely unknown.

The hypothalamic–pituitary–adrenal (HPA) axis, the primary regulator of human endogenous cortisol secretion, is a potential biological mediator between early life adversity and later adverse neurodevelopmental and cardiometabolic phenotypes. Its candidacy is supported by a growing body of preclinical and human observation research that demonstrates a complex interplay between early life adversity, adaptations in cortisol regulation and health across the life course.^6^ Epidemiological studies commonly use saliva cortisol, which has been validated as a non-invasive marker of serum cortisol concentrations through many studies.^7^

Previous studies of infant HPA axis regulation after preterm birth have typically focused on stress reactivity. There is emerging evidence that infants born preterm may have a blunted cortisol response to physiological and psychological stressors^8 9^ and that this potentially relates to the degree of prematurity^10^ and sex.^11^

To date, only one study has assessed basal cortisol of term and preterm infants across infancy. Infants born between 28 and 32 weeks (very preterm) and <28 weeks (extremely preterm) had lower cortisol concentrations than term infants at 3 months corrected age, with extremely preterm infants having higher levels at 8 months.^12^ Assessments were made using single saliva collections at each timepoint, which is unlikely to be representative of cortisol output across the day. Additionally, single measurements do not allow for characterisation of diurnal rhythmicity. In adults cortisol concentrations typically peak in the morning before falling across the day, and the extent of this decline can be described as the diurnal slope.

This study presents data from a prospective birth cohort conducted with the primary aim of investigating whether preterm birth predisposes infants to differences in stress-induced and diurnal cortisol release in infancy. We hypothesised preterm birth would predispose infants to a blunted stress response to vaccination and flatter decline in cortisol across the day.^13 14^ Given the complications of preterm birth are gestation-specific,^15^ with adverse effects more likely for infants born at younger gestations, we hypothesised HPA axis programming effects would be most pronounced in infants born extremely preterm. Additionally, we sought to assess if HPA axis programming after preterm birth was sex-specific.

## Methods

### Participants

The ‘Stress Response Systems in Mothers and Preterm Infants’ study recruited infants born at the Royal Infirmary of Edinburgh, UK, between March 2018 and August 2019. All parents gave written informed consent. Exclusion criteria were regular maternal steroid use in pregnancy and congenital chromosomal or structural abnormalities in infants.

At 4 months chronological age infants had four saliva samples collected at the time of routine scheduled vaccinations against diphtheria, tetanus, pertussis, polio, haemophilus influenzae type b and hepatitis B (DTaP/IPV/Hib/HepB), meningitis B and pneumococcus (13 serotypes). Vaccinations were administered as three intramuscular injections at participants’ general practitioner surgeries, as per routine clinical care. Appointments were attended by a member of the research team, who collected samples immediately before and 20 min after vaccinations, assessing cortisol reactivity. Morning (07:30–09:30) and evening (19:30–21:30) samples were collected by participants’ parents in their own homes, assessing diurnal cortisol regulation. Parents were asked to collect these samples on a single day in the week preceding the vaccination and to refrigerate samples before transferring them to the research team at the vaccination appointment. Saliva was collected using Salimetrics SalivaBio Infant Swabs, stored at −80°C and analysed by liquid chromatography tandem mass spectrometry (LC-MS/MS) at Edinburgh University Clinical Research Facility Mass Spectrometry Core (online supplemental appendix 1).

10.1136/fetalneonatal-2021-321593.supp1 Supplementary data

### Statistical analysis

**Figure 1:**
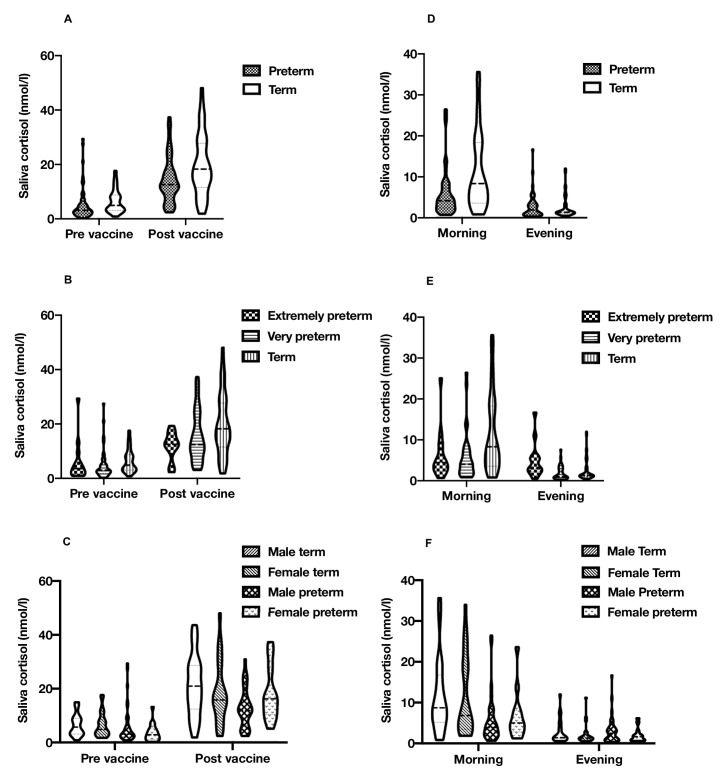
Violin plots of saliva cortisol levels. Range, median and quartiles for (A) term/preterm vaccine response (n=45 vs 56); (B) term/extremely/very preterm vaccine response (n=45 vs 42 vs 14); (C) sex/birth group vaccine response (n=21 vs 24 vs 38 vs 18); (D) term/preterm diurnal cortisol (n=42 vs 48); (E) term/extremely/very preterm diurnal cortisol (n=42 vs 34 vs 14); and (F) sex/birth group diurnal cortisol (n=20 vs 22 vs 35 vs 13). The figure was produced by DQS using GraphPad Prism V.8.4.3.

Analyses were performed using IBM SPSS Statistics V.25. Continuous data are summarised as mean±SD if normally distributed and median (IQR) if skewed. One participant concurrently using topical steroids was excluded from analysis. One morning cortisol sample was also excluded (as it had a supraphysiological cortisol concentration at >50 nmol/L). Distributions of cortisol concentrations were assessed for normality using histograms. Cortisol concentrations for the assessment of diurnal cortisol regulation were positively skewed and log_10_-transformed for further analysis. Prevaccine cortisol concentrations were positively skewed. However, vaccine cortisol concentrations were not transformed in the presented analysis as postvaccine cortisol concentrations and subsequent regression model residuals showed only slight asymmetry. Cortisol distributions across all timepoints are presented through violin plots in figure 1.

Comparisons of HPA axis regulation between term and preterm infants (defined as a single group) and preterm subgroups (defined as extremely preterm birth at <28 weeks or very preterm birth at 28–32 weeks gestation) were conducted using linear mixed models. Cortisol reactivity in response to vaccination and diurnal cortisol patterns (average cortisol across the day and diurnal slope) were tested separately. In each model, time (defined as prevaccination=0 and postvaccination=1, or evening=0 and morning=1) was specified as a repeated measure. Preterm birth’s influence on cortisol reactivity and diurnal cortisol decline across the day was tested by the time*preterm/term group interaction. To test if HPA axis programming is limited, or differs by degree of prematurity, the time*extremely preterm/very preterm/term interaction was assessed. To assess if associations between preterm birth and HPA axis regulation differed by sex, the time*sex*preterm/term interaction was tested. This process was also undertaken for assessment of mean diurnal cortisol levels, using morning and evening cortisol concentrations in models that did not include an interaction with time.

All analyses were conducted with models specifying a diagonal covariance structure (assuming no association between and unequal variance of the repeated measures). This is because the variance of cortisol concentrations at the different timepoints differed, and models incorporating a correlation in cortisol concentrations between participant samples did not improve the model fit assessed through comparison of −2 log likelihood ratios.

Sensitivity analyses were conducted assessing the potential effect of comparing infants at chronological compared with corrected ages. In this study, where saliva was collected at 4 months chronological age, the corrected age of saliva samples collected in the morning and evening (Pearson’s correlation coefficient, r=0.908) and around the vaccination (r=0.876) was strongly correlated with birth gestation. As corrected age at saliva sampling could not directly be adjusted for in models without introducing multicollinearity, the potential influence of corrected age at sampling on cortisol concentration was assessed through univariate analysis in term and preterm groups separately.

Effect sizes are reported as mean differences in cortisol concentrations for normally distributed data and percentage differences of geometric means for skewed data, with 95% CI. P<0.05 was considered statistically significant.

## Results

### Demographics

Infant and sampling demographics are presented in table 1. As sample collection was timed according to when infants received their third set of routine vaccinations (and the vaccine schedule is scheduled according to postnatal age), corrected age at sampling differed between birth groups. Cortisol reactivity to vaccination was assessed at a median of 5.6 weeks corrected age (IQR 3.7–11.8) in extremely preterm infants and 9.0 weeks (IQR 8.1–11.8) in very preterm infants.

**Table 1:** Study demographics

	Extremely preterm (≤27+6)	Very preterm (28+0–32+0)	Term (≥37+0)
Infant characteristics			
Number of participants*	16	42	45
Birth gestation (weeks)	26.4 (25.2–27.2)	30.8 (29.5–31.3)	40.1 (39.2–41.0)
Male, n (%)	12 (75)	28 (67)	21 (47)
Female, n (%)	4 (25)	14 (33)	24 (53)
Birth weight (g)	909±219	1436±388	3556±437
Birthweight z-score†	0.3±0.7	−0.2±1.3	0.5±1
Singleton, n (%)	12 (75)	30 (71)	45 (100)
Twin, n (%)	4 (25)	12 (29)	0 (0)
Sampling characteristics			
Chronological age at diurnal samples (weeks)	19.9 (17.3–23.5)	18.4 (16.9–21.1)	17.1 (16.3–18.9)
Corrected age at diurnal samples (weeks)	5.4 (4.0–8.9)	8.9 (7.9–11.2)	
Chronological age at vaccine (weeks)	20 (16.8–25.6)	18.8 (17.3–20.8)	17.1 (16.4–18.7)
Corrected age at vaccine (weeks)	5.6 (3.7–11.8)	9.0 (8.1–11.8)	
Time of day at morning sample	08:45 (08:08–09:48)	08:42 (08:06–09:20)	08:23 (07:50–09:05)
Time of day at evening sample	20:15 (19:40–21:11)	20:19 (19:46–21:19)	19:52 (19:34–20:30)
Time of day at vaccine	11:37 (11:14–13:35)	11:31 (10:34–13:36)	11:03 (10:05–13:21)

Continuous data are presented as mean±SD if normally distributed and median (IQR) if skewed.

*Data were unavailable for 2 participants around vaccination and 13 for diurnal assessments due to missed or insufficient volume collections.

†Birthweight z-scores were calculated as per the International Fetal and Newborn Growth Consortium for the 21st Century (INTERGROWTH) standards.

### Cortisol response around vaccination

Salivary cortisol increased in response to vaccination in all groups (p<0.001) (table 2). Compared with infants born at term, no difference in cortisol reactivity was observed in the combined preterm group (−3.9 nmol/L, p=0.09) (figure 1A) or in very preterm infants (−2.7 nmol/L, p=0.26) (figure 1B). In contrast, cortisol reactivity in response to vaccination was blunted in extremely preterm infants (−7.3 nmol/L, p=0.03). There was also an interaction between birth group and sex (p=0.02). Male preterm infants had a blunted cortisol response compared with male term infants (−7.7 nmol/L, p=0.009), but female infants did not show this pattern of response (2.5 nmol/L, p=0.47) (figure 1C).

**Table 2:** Cortisol reactivity to vaccination

Birth group	Cortisol reactivity to vaccine Mean±SD	Estimated group differences in cortisol reactivity (95% CI)	P value
Birth group comparisons			
Term (≥37 weeks), n=45	13.1±11.9	Reference	
Preterm (≤32 weeks), n=56	9.3±10.2	−3.9 (−8.3 to 0.5)	0.09
Extremely preterm (<28 weeks), n=14	5.8±8.5	−7.3 (−14.0 to −0.6)	0.03
Very preterm (28–32 weeks), n=42	10.4±10.6	−2.7 (−7.4 to 2.0)	0.26
Birth group comparisons stratified by sex			
Male term (≥37 weeks), n=21	14.5±11.7	Reference	
Male preterm (≤32 weeks), n=38	6.8±8.7	−7.7 (−13.4 to −2.0)	0.009
Female term (≥37 weeks), n=24	11.9±12.2	Reference	
Female preterm (≤32 weeks), n=18	14.5±11.5	2.5 (−4.5 to 9.6)	0.47

Cortisol reactivity was calculated by subtracting the prevaccine cortisol concentration from the postvaccine cortisol concentration.

Effect sizes represent cortisol concentrations in nmol/L.

### Diurnal cortisol

At 4 months chronological age, cortisol concentrations reduced across the day, with higher cortisol in the morning than evening (p<0.001) (table 3). Compared with infants born at term, preterm infants had a reduced mean cortisol across the day (−26.8%, p=0.02) and a flattened diurnal slope (−48.8%, p=0.01) (figure 1D). In comparison with term infants, extremely preterm infants had a flattening of diurnal slope (−72.9%, p<0.001) but no difference in mean concentrations across the day (13.3%, p=0.52), with very preterm infants having lower mean levels (−36.6%, p=0.002), but not a significant flattening of the diurnal slope (−33.6%, p=0.15) (figure 1E). In a post-hoc analysis conducted to interrogate these differing patterns of diurnal cortisol observed between the preterm groups, extremely preterm (p=0.04) and very preterm (p=0.002) infants both had lower morning cortisol concentrations than term infants, while only infants born extremely preterm (p=0.006) had higher evening cortisol concentrations. Sex did not interact with birth group and when testing the associations for diurnal slope (p=0.78) or mean levels across the day (p=0.62) (figure 1F).

**Table 3:** Diurnal cortisol

Birth group	Morning cortisol	Evening cortisol	Mean daily cortisol			Diurnal slope		
	Geometric mean±SD*	Geometric mean±SD*	Geometric mean±SD*	Estimate of group effect Percentage (95% CI)†	P value	Geometric mean±SD‡	Estimated group differences in cortisol slope Percentage (95% CI)†	P value
Term (≥37 weeks), n=42	7.8±2.6	1.7±2.3	3.6±1.8	Reference		4.6±3.9	Reference	
Preterm (≤32 weeks), n=48	4.1±2.4	1.7±2.6	2.7±1.8	−26.8 (−44.1 to −4.3)	0.02	2.3±3.9	−48.8 (−69.8 to −13.2)	0.01
Extremely preterm (<28 weeks), n=14	4.3±2.4	3.5±2.4	3.9±1.7	13.3 (−22.8 to 66.3)	0.52	1.2±3.8	−72.9 (−87.1 to −42.8)	<0.001
Very preterm (28–32 weeks), n=34	4.0±2.4	1.3±2.3	2.3±1.8	−36.6 (−52.4 to −15.6)	0.002	3.0±3.6	−33.6 (−62.0 to 16.0)	0.15

Analyses were conducted using log_10_-transformed cortisol values.

*Mean morning, evening and daily log_10_ cortisol values were back-transformed producing geometric means.

†Back-transformed regression coefficients represent percentage differences in cortisol metrics between groups.

‡ Diurnal slope representing the ratio of morning and evening geometric means was calculated through back transformation of mean log_10_ (morning / evening) cortisol values.

### Sensitivity analyses

Mean diurnal cortisol concentrations were negatively associated with corrected age at sampling in term (p=0.02) but not in preterm (p=0.73) infants. Cortisol diurnal slope and reactivity to vaccination were not associated with corrected age at sampling in either the term or preterm group (online supplemental table 1).

## Discussion

This study has two main findings. First, extremely preterm birth is associated with altered HPA axis regulation, including both blunted stress response and flattened diurnal slope in infancy. Second, infant sex was related to HPA axis programming of cortisol reactivity but not diurnal cortisol concentrations.

The ‘developmental origins of health and disease’ (DOHaD) hypothesis postulates that environmental exposures in early life, during periods critical for development, can influence health across the life course.^16^ Interest in this concept has expanded since it was shown that reduced fetal size, a surrogate for an adverse in utero environment, is associated with insulin resistance^17^ and cardiovascular disease in adulthood.^18^

At 4 months chronological age extremely preterm infants had a reduced cortisol response to vaccination and a reduced decline in cortisol across the day. While our primary hypothesis was that infants born ≤32 weeks gestation would show adaptation of the HPA axis, an increased propensity for HPA axis programming in infants born extremely preterm appears physiologically plausible, when viewed through a DOHaD lens, as this group face an earlier disruption to environment and greater morbidity in the newborn period. Additionally, evidence from previous studies provides precedent that HPA axis programming may be gestation-specific.^10 12^

This study adds to evidence that early exposure to an ex utero environment contributes to blunted cortisol reactivity across life.^8–10 19 20^ Blunting of cortisol reactivity in male compared with female preterm infants is consistent with a previous observation of cortisol reactivity of preterm infants in response to vaccination^11^ and adds to evidence of vulnerability of the male axis after preterm birth.^21–23^

This study is the first to address whether preterm birth influences diurnal slope in infancy. However, available data from studies in childhood are indicative of a flattening of the HPA axis after preterm birth, including two reports of reduced morning cortisol^24 25^ and one of increased evening cortisol concentrations.^23^ Furthermore, a flatter diurnal cortisol slope and blunted cortisol reactivity to stressors are patterns of HPA axis regulation frequently observed after childhood adversity.^13 14 26^

The altered patterns of saliva cortisol observed in this study are of clinical interest as HPA axis dysregulation is potentially causative of cardiometabolic^27^ and neurobehavioural pathology.^28^ Importantly evidence is emerging that flattening of the diurnal slope after early life stress is reversible, and so this may be a target for future intervention.^29^

Multiple perinatal exposures hold the potential to contribute to programming of the HPA axis in a preterm population. These include intrauterine and extrauterine nutritional deficiencies and growth restriction,^30^ chorioamnionitis and postnatal septic episodes,^31^ and noxious environmental stimuli.^21 32^

Additionally, perinatal hormonal exposures could change the way that cortisol secretion is regulated at the level of the hypothalamus or pituitary glands across infancy, or alter the development of the adrenal cortex. Preterm infants are commonly exposed to exogenous steroids and higher endogenous cortisol concentrations than would be expected at a comparative gestation in utero,^33^ and a blunted stress response and flattened diurnal cortisol could reflect adaptations of the HPA axis in response to these exposures.

Preterm infants also have reduced exposure to hormones typically secreted during the third trimester by the placenta, such as reduced corticotropin-releasing hormone, oestrogen and kisspeptin exposure. Reduced exposure to these hormones likely changes the developmental trajectory of the adrenal gland,^34 35^ an organ that undergoes considerable remodelling after birth with reduction in the androgen-producing fetal zone and maturation of the cortisol-producing zona fasciculata.

This study’s primary strength is that it combines assessment of both cortisol stress response and diurnal rhythm, providing an indepth characterisation of the preterm HPA axis in infancy. Additionally cortisol concentrations were quantified using LC-MS/MS using a long 16 min chromatographic analysis, such that temporal separation confidently excludes contribution by cortisone and corticosterone and its 11-dehydrocorticosterone, while distinct mass differences of cortisol (~362 Da) and androgens (~290 Da) allow quantification without interference by other adrenal steroids.

A potential limitation is that sampling was scheduled according to chronological age and so the corrected ages of participants in the extremely preterm group were younger than those at sampling of term group. There has been disagreement around whether adrenal function relates to postmenstrual age or time after birth, particularly around the involution of the fetal zone and continued production of fetal zone steroids.^34 36^ However, it has previously been demonstrated that both preterm and term-born infants typically have a higher morning than evening cortisol from 1 month corrected age, suggesting that corrected age may be more important for diurnal cortisol rhythms.^37 38^ Thus the younger gestational age of sampling in the cohort may have contributed to the flatter diurnal cortisol rhythms observed in extremely preterm compared with term-born infants. In contrast, it is unlikely that assessment at a younger corrected age contributed to the reduced cortisol response to vaccination observed in extremely preterm infants compared with term infants, as previous longitudinal studies of stress reactivity in infancy have demonstrated reductions, rather than increases, in cortisol reactivity with progressing age.^33 39^

Another limitation is that cortisol concentrations before the vaccination were higher than morning cortisol concentrations in the preterm groups. This suggests that attending the vaccination appointment may itself be a mild stressor. Additionally, the sample sizes of the extremely preterm group and female infants born preterm were relatively small, reducing the precision of effect size estimates for these groups.

Preterm birth has also been associated with adaptations in adrenal androgen secretion from the postnatal period into young adulthood,^40 41^ and these adaptations in turn hold further potential to influence neurodevelopmental and metabolic phenotypes. Future studies incorporating the measurement of both cortisol and adrenal androgens would enable a more complete understanding of adrenal function after preterm birth.

## Conclusion

Extremely preterm birth disrupts the normative development of the HPA axis in infancy, with patterns of cortisol secretion resembling those seen after childhood adversity. Future research needs include testing how HPA axis adaptation relates to adverse neurodevelopmental and metabolic phenotypes seen after preterm birth and assessing whether psychosocial interventions can ‘reprogramme’ the preterm HPA axis.
