# Supplementary material for: Saliva cortisol diurnal variation and stress responses in term and preterm infants
Source: Arch Dis Child Fetal Neonatal Ed. 2022 Mar 7;107(5):558–64. doi: 10.1136/archdischild-2021-321593 (PMC9411886; doi:10.1136/archdischild-2021-321593)
Supplement: Supplementary data [file fetalneonatal-2021-321593supp001.pdf]

Supplementary Materials

Title:

Preterm birth and infant hypothalamic-pituitary-adrenal axis regulation

Contains:

Supplementary Appendix 1: Liquid chromatography tandem mass spectrometry (LC-MS/MS) methodology for steroid hormone analysis

Supplementary Table 1. Associations of diurnal cortisol regulation with the corrected gestational age at sampling, Edinburgh study

Supplementary Appendix 1: Liquid chromatography tandem mass spectrometry (LC-MS/MS) methodology for steroid hormone analysis

Saliva (100 μL) was enriched with 9,11,12,12-[$^{2}H_{4}$]-cortisol (d4F) (2.5 ng) as internal standard, and prepared by automated extraction on a Biotage Extrahera liquid handling robot, using ISOLUTE®, SLE+ 200 Supported Liquid Extraction (Biotage, Uppsala, Sweden), eluting with dichloromethane/isopropanol (95:5; 1 mL). Extracts were prepared alongside a calibration curve of cortisol (Cerilliant/Sigma-Aldrich, Dorset, UK). The salivary extract was analysed by LC-MS/MS on a Shimadzu Nexera X2 and a 6500+ QTrap (Sciex, Warrington, UK), by separation on a Kinetex C18 (150 x 3 mm; 2 μm; Phenomenex, UK) column using a water/methanol mobile phase containing 0.05 mM ammonium fluoride flowing at 0.3 mL/min at 40ºC over 16 minutes, adapted from Denham et al (1). Cortisol eluted at 4 mins and was temporally separated from cortisone (3.3 mins) and corticosterone (5.1 mins) ensuring no interference from isotopologues or common product ions. The mass spectrometer was controlled and data acquired using Sciex Analyst® 1.6.3 Software. Data were integrated using Quantitate. The mass spectrometer was operated in positive ion electrospray ionisation using a TurboIonSpray source (600ºC, 5,5 kV) and data collected in unit resolution (0.7 m/z full width at half maximum). The method monitored for *m/z* 363 - 121 and 91 for cortisol and *m/z* 367 - 121 and 91 for d4F with a scan time of 20 milliseconds, calculating the peak area ratio of cortisol to the internal standard, d4F, and using linear regression with 1/x weighting of a calibration curve covering the range 0.015 – 100 ng/mL (Inter-assay Precision 4.1% RSD, bias 5.8%).

Supplementary Table 1. Associations of diurnal cortisol regulation with the corrected gestational age at sampling

Group	Mean daily cortisol		Diurnal slope		Cortisol reactivity to vaccination	
	Percentage (95% CI) [b]	p-value	Percentage (95% CI) [b]	p-value	Concentration (95% CI) [a]	p-value
Term	-13.8 (-24.1, -2.1)	0.02	9.7 (-15.2, 41.8)	0.48	0.2 (-2.0, 2.5)	0.83
Preterm	-1.1 (-7.0, 5.2)	0.73	2.5 (-9.3, 16.0)	0.69	-0.3 (-1.2, 0.6)	0.54

[a] Regression coefficients represent change in cortisol reactivity to vaccination in nmol/L with each corrected week of age at sampling. [b] Diurnal cortisol metrics were analysed through $\log_{10}$-transformed data. Back-transformed regression coefficients, represent percentage differences in these metrics with each additional corrected week of age at sampling.

References

1. Denham SG, Just G, Kyle C.J, *et al*. Automated Supported Liquid Extraction for the analysis of a panel of 12 endogenous steroids in human plasma by LC-MS/MS. *Preprints* 2020 (doi: 10.20944/preprints202011.0551.v1).
